# Immunohistochemistry-based risk stratification of upper tract urothelial carcinoma

**DOI:** 10.3389/fonc.2026.1797142

**Published:** 2026-03-24

**Authors:** Dong-Lin He, Guo-Run Zi, Da-Jiang Zhang, Huan-Qiao Zhong, Chen Wang, Run-Lin Feng, Chang-Xing Ke

**Affiliations:** 1 Department of Urology, The Second Affiliated Hospital of Kunming Medical University, Kunming, China; 2 Department of Pathology, The Second Affiliated Hospital of Kunming Medical University, Kunming, China

**Keywords:** immunohistochemistry, molecular subtyping, nomogram, prognosis, upper tract urothelial carcinoma

## Abstract

**Background:**

Upper tract urothelial carcinoma (UTUC) is characterized by significant biological heterogeneity. Although high-throughput sequencing has unveiled the genomic landscape of UTUC, its routine clinical application is hindered by high costs and technical complexity. This study aimed to develop a cost-effective molecular subtyping strategy using immunohistochemistry (IHC) as surrogate markers and to construct a novel risk stratification model integrating driver genes (*TP53/FGFR3*) for predicting postoperative recurrence-free survival (RFS).

**Methods:**

We retrospectively analyzed 215 patients with UTUC who underwent radical nephroureterectomy between 2021 and 2024. IHC staining was performed for GATA3, CK20, CK5/6, CD44, P53, and FGFR3. Tumors were initially stratified into Luminal-like or Basal-like subtypes. Subsequently, a three-tier “RiskGroup” stratification was constructed: Low Risk (Luminal-like with wild-type P53), Intermediate Risk (Luminal-like with aberrant P53), and High Risk (Basal-like). A prognostic nomogram was developed based on multivariate Cox regression analysis and validated using the Concordance Index (C-index) and Decision Curve Analysis (DCA).

**Results:**

Luminal-like and basal-like subtypes showed distinct clinicopathologic profiles. RiskGroup further captured heterogeneity, with increasing Ki-67 across low-, intermediate-, and high-risk groups (median 30%, 60%, 60%; P<0.001). The intermediate-risk group, despite luminal immunophenotype, was enriched for high-grade disease (94.2%) and had worse RFS than the low-risk group. RFS differed across RiskGroup (P<0.001); the high-risk group had the poorest outcomes and remained independently associated with RFS (HR 2.60, 95% CI 1.27–5.33; P = 0.009), whereas intermediate risk was not significant after adjustment (HR 1.63; P = 0.227). RiskGroup, pathological T stage, and lymphovascular invasion were independent prognostic factors. The nomogram showed good discrimination (C-index 0.769) and 1-year RFS AUC 0.823 with net clinical benefit.

**Conclusion:**

This IHC-based, biologically informed stratification identifies an occult aggressive subset within luminal tumors and complements routine pathology. The internally validated nomogram is exploratory given cohort composition and lack of external validation; multicenter validation is warranted.

## Introduction

Upper tract urothelial carcinoma (UTUC) is a relatively rare malignancy globally. According to data from the European Association of Urology (EAU) and international multicenter studies, UTUC accounts for only 5%–10% of all urothelial carcinomas (1, 2). Currently, the clinical management of UTUC is evolving from surgery alone towards a multimodal treatment paradigm. Although radical nephroureterectomy (RNU) remains the gold standard (1), therapeutic options have expanded to include platinum-based adjuvant chemotherapy, immune checkpoint inhibitors (e.g., PD-1/PD-L1 inhibitors) (3), and kidney-sparing surgery (KSS) for selected low-risk patients (4). However, clinical practice is frequently challenged by the significant biological heterogeneity of UTUC. Patients with identical TNM stages and pathological grades often exhibit vastly divergent clinical trajectories and sensitivities to adjuvant therapies (5, 6). This intra-stage heterogeneity suggests that traditional clinicopathological parameters are no longer sufficient to meet the demands of precision oncology.

With the advent of the precision medicine era, elucidating the intrinsic molecular mechanisms of tumors is essential for identifying distinct patient subgroups. While the genomic landscape of bladder cancer has been extensively explored, providing references for diagnosis, prognosis, and therapeutic targets (7), the molecular characteristics of UTUC do not entirely mirror those of urothelial carcinoma of the bladder (UCB) (8). Furthermore, most existing molecular studies are derived from Western populations. Given the distinct epidemiological factors (e.g., aristolochic acid exposure) associated with UTUC in Asia, there remains a paucity of research regarding the specific molecular profiles and pathogenic mechanisms in the Chinese population (9–11).

Although next-generation sequencing (NGS) can provide a comprehensive molecular atlas, its widespread implementation in routine clinical pathology is hindered by prohibitive costs, long turnaround times, and the lack of specialized bioinformaticians and standardized analysis pipelines within most clinical centers (12). Consequently, there is an imperative need to explore “clinical versions” of molecular subtyping using cost-effective, accessible, and standardized immunohistochemistry (IHC) techniques. Multiple studies have confirmed that IHC-based subtyping using a panel of 2–4 core antibodies achieves 85%–90% concordance with transcriptome-based classifications, and its predictive value has been validated in independent Western cohorts such as MDA and Lund (13, 14). However, large-scale studies validating these IHC surrogate markers for risk stratification specifically in Chinese UTUC patients are still lacking. This study aims to bridge this gap by establishing an IHC-based molecular subtyping system and a novel prognostic model to provide robust evidence for individualized clinical decision-making.

## Materials and methods

### Patient cohort

This retrospective study was approved by the Ethics Committee of the Second Affiliated Hospital of Kunming Medical University (Approval No. Shen-PJ-Ke-2025-193). The requirement for informed consent was waived due to the retrospective nature of the study. We analyzed data from a cohort of patients with UTUC who underwent radical nephroureterectomy (RNU) with bladder cuff excision at the Department of Urology between January 2021 and December 2024. The inclusion criteria were: (1) histologically confirmed urothelial carcinoma; (2) availability of formalin-fixed paraffin-embedded (FFPE) tissue blocks for immunohistochemical (IHC) analysis; and (3) complete clinicopathological and follow-up data. Patients were excluded if they had: (1) received neoadjuvant chemotherapy, radiotherapy, or immunotherapy (i.e., treatment-naïve status); (2) a history of other malignancies; (3) non-urothelial histology (e.g., pure squamous cell carcinoma or adenocarcinoma); or (4) insufficient tissue for analysis. Tumor staging was assessed according to the 8th edition of the AJCC/UICC TNM classification system, and histological grading was determined based on the 2004/2016 WHO classification system.

### Immunohistochemistry analysis

IHC staining was performed on 4-μm-thick FFPE tissue sections using the standard Streptavidin-Peroxidase (SP) method. The panel consisted of four molecular subtyping markers (GATA3, CK20, CK5/6, and CD44) and two prognostic markers (P53 and FGFR3). Automated staining was conducted using the [Celnovte CNT360-M2] stainer (Celnovte Biotechnology Co., Ltd., Henan, China). The primary antibodies and their dilution ratios were as follows: GATA3 ([EP368, MXB Biotechnologies], 1:100), CK20 ([MX059, MXB Biotechnologies], 1:100), CK5/6 ([MX040, MXB Biotechnologies], 1:100), CD44 ([2F10, MXB Biotechnologies], 1:100), P53 ([MX008, MXB Biotechnologies], 1:100), and FGFR3 ([1F3G1, Proteintech Group], 1:500). Antigen retrieval was performed using citrate buffer (pH 6.0) or EDTA (pH 9.0) depending on the specific antibody requirements.

**Figure 1:**
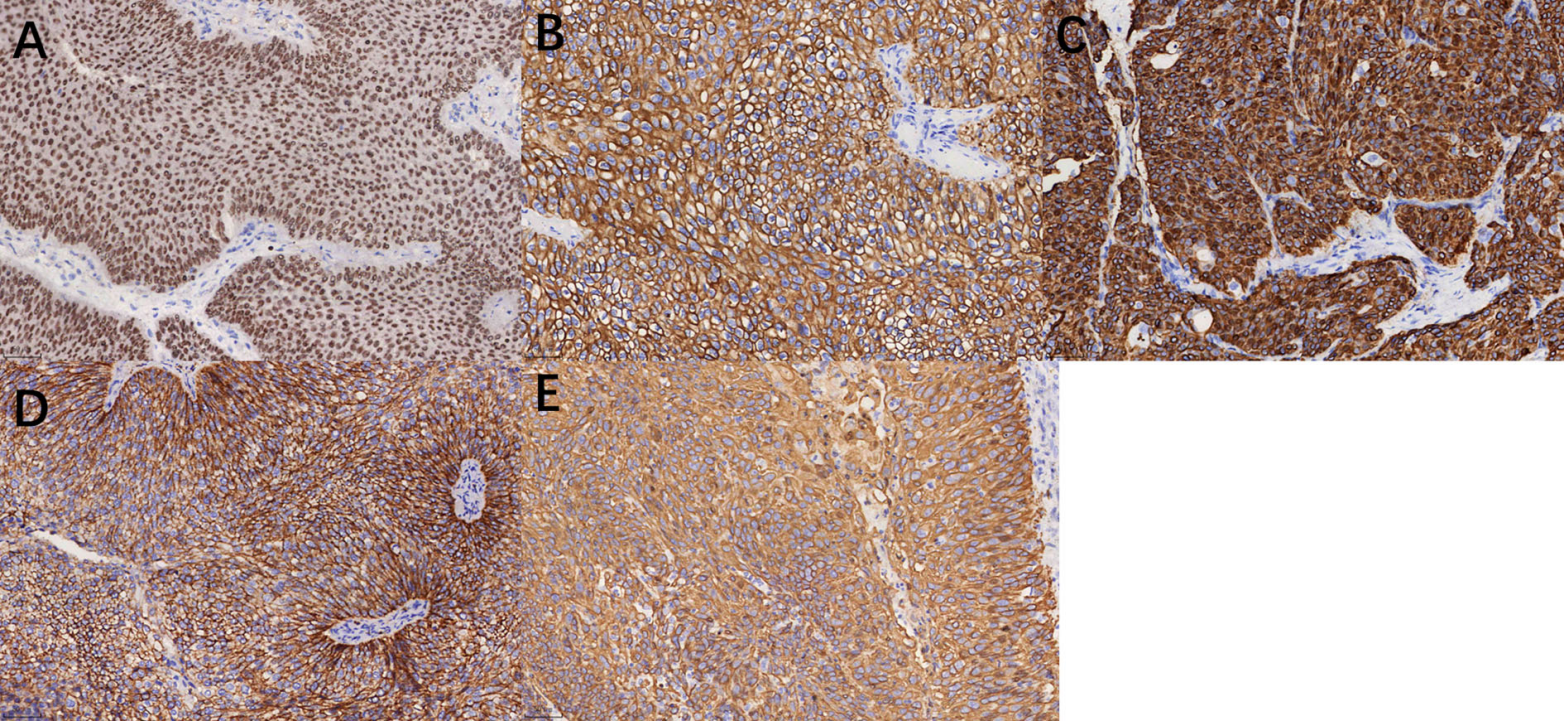
Immunohistochemical staining of markers (×200). **(A)** GATA3 positive; **(B)** CK20 positive; **(C)** CK5/6 positive; **(D)** CD44 positive; **(E)** FGFR3 positive.

**Figure 2:**
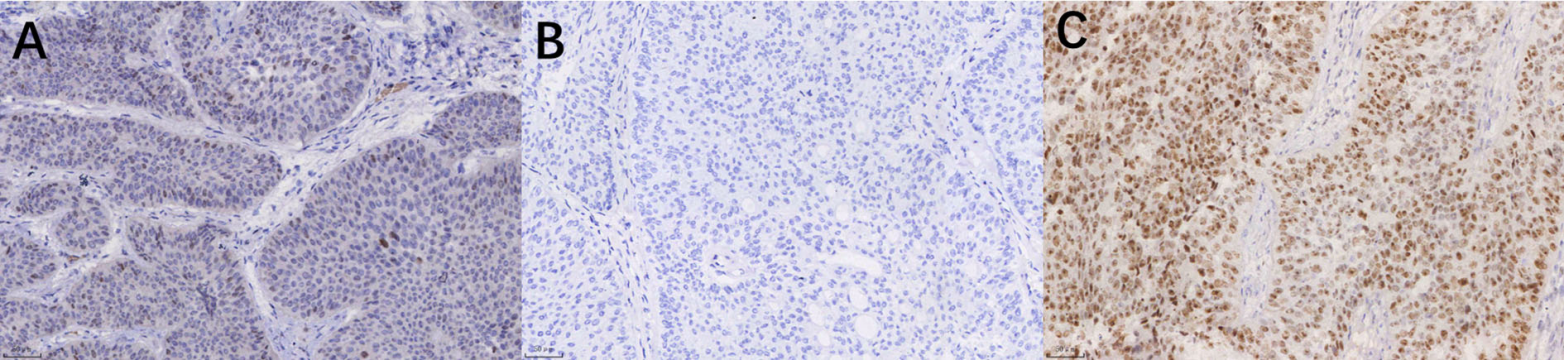
Immunohistochemical expression patterns of P53 (Original magnification ×200). **(A)** Wild-type pattern; **(B)** Mutant-type pattern (Complete absence/Null type); **(C)** Mutant-type pattern (Diffuse strong positivity).

Slides were evaluated independently by two senior pathologists blinded to the patients’ clinical outcomes, and any discrepancies in scoring were resolved through joint review to reach a consensus. A semi-quantitative Immunoreactive Score (IRS) was calculated by multiplying the staining intensity score (0: negative; 1: weak; 2: moderate; 3: strong) by the percentage of positive cells score (0: 0%; 1: ≤10%; 2: 11%–50%; 3: 51%–75%; 4: >75%), yielding a total score ranging from 0 to 12. Cut-off Definition: For GATA3, CK20, CK5/6, CD44, and FGFR3, based on the study by Dadhania et al. (15), a total score of ≥2 was defined as Positive, while a score of 0–1 was defined as Negative.(**Figure 1**) P53 Interpretation: P53 expression was categorized as Mutant-type (indicating complete absence or diffuse strong positivity in >50% of cells) or Wild-type (heterogeneous, weak-to-moderate staining in 1%–50% of cells) (16) (**Figure 2**).

### Subtyping strategy

We adopted a hierarchical strategy using IHC as surrogate markers for intrinsic molecular subtypes, adapted from the approaches of Rodriguez et al. (17) and Zhou et al. (18). First, a two-tier subtyping was constructed based on core marker expression: tumors were defined as Luminal-like if the sum of luminal marker scores (GATA3, CK20) exceeded that of basal markers (CK5/6, CD44); conversely, tumors were classified as Basal-like if the sum of basal marker scores was greater than or equal to that of luminal markers, or if all four markers were negative (double-negative). Subsequently, to optimize prognostic evaluation, we integrated the status of the driver gene P53 to construct a three-tier “RiskGroup” stratification model: Low Risk: Luminal-like tumors with Wild-type P53 (representing the classical luminal-papillary pathway); Intermediate Risk: Luminal-like tumors with Mutant-type P53 (representing the luminal-infiltrated or genomically unstable pathway); High Risk: All Basal-like tumors (representing the most aggressive biological phenotype).

### Statistical analysis

Statistical analyses were performed using SPSS software version 27.0 (IBM Corp., Armonk, NY, USA) and R software version 3.6.2. Categorical variables were compared using the Chi-square test or Fisher’s exact test. Overall survival (OS) and recurrence-free survival (RFS) were estimated using the Kaplan-Meier method and compared via the Log-rank test. Univariate and multivariate Cox proportional hazards regression models were used to identify independent prognostic factors, presenting Hazard Ratios (HR) with 95% Confidence Intervals (CI). A nomogram was established based on the multivariate analysis results. The model’s predictive performance was assessed using the Concordance Index (C-index), calibration curves, Time-dependent ROC curves, and Decision Curve Analysis (DCA). A two-sided P-value < 0.05 was considered statistically significant.

## Results

### Clinicopathological characteristics

A total of 215 patients with UTUC were included in this study. The median age of the cohort was 67.0 years (IQR: 59.0–73.0), with a male predominance (65.6%). Tumor locations were distributed as follows: renal pelvis (44.7%), ureter (30.2%), and multifocal sites (25.1%). Histopathological evaluation revealed a significant malignancy burden; high-grade tumors accounted for 80.0% of cases, and locally advanced disease (pT3) was the most prevalent pathological stage (43.7%). Furthermore, lymphovascular invasion (LVI) was identified in 35.8% of patients, while 17.2% exhibited histological variants. The median Ki-67 proliferation index was 50.0% (**Table 1**).

**Table 1:** Baseline clinicopathological characteristics of 215 patients with UTUC.

Characteristics	Subgroup	No. (%)
Age (years)	Median (IQR)	67(59–73)
Gender	Male	141(65.6)
	Female	74(34.4)
Smoking history	Yes	69(32.1)
	No	146(67.9)
Tumor location	Renal pelvis	96(44.7)
	Ureter	65(30.2)
	Multifocal	54(25.1)
Pathological T stage	Ta/T1	78(36.3)
	T2	33(15.3)
	T3	94(43.7)
	T4	10(4.7)
Tumor grade	High grade	172(80.0)
	Low grade	43(20.0)
Ki-67 index (%)	Median (IQR)	50.0(30.0-70.0)
LVI	Yes	77(35.8)
	No	138(64.2)
Concomitant CIS	Yes	16(7.4)
	No	199(92.6)
Histological variant	Yes	37(17.2)
	No	178(82.8)

### Two-tier molecular subtyping and prognostic analysis

**Figure 3:**
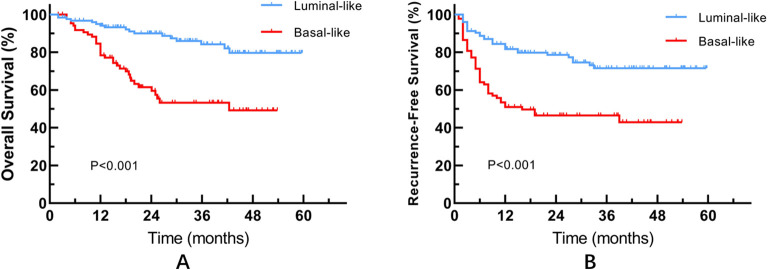
Kaplan-Meier survival curves based on the two-tier molecular subtyping. **(A)** Overall Survival (OS); **(B)** Recurrence-Free Survival (RFS).

Based on the expression profiles of GATA3/CK20 and CK5/6/CD44, patients were stratified into Luminal-like (*n* = 126, 58.6%) and Basal-like (*n* = 89, 41.4%) subtypes. Significant clinicopathological disparities were observed between the two groups (**Table 2**). The Basal-like subtype displayed a markedly more aggressive phenotype compared to the Luminal-like subtype, characterized by a significantly higher proportion of locally advanced tumors (pT3/pT4) (69.7% vs. 33.3%, *P* < 0.001), LVI positivity (48.3% vs. 27.0%, *P* = 0.001), and variant differentiation (31.5% vs. 7.1%, *P* < 0.001). Kaplan-Meier survival analysis demonstrated that patients with the Basal-like subtype experienced significantly inferior Overall Survival (OS) and Recurrence-Free Survival (RFS) (Log-rank *P* < 0.001) (**Figure 3**). Multivariate Cox regression analysis further confirmed that the Basal-like subtype was an independent predictor of poor RFS (HR = 1.932, 95% CI: 1.169–3.194, *P* = 0.01), independent of pathological T stage and LVI (**Table 3**).

**Table 2:** Comparison of clinicopathological characteristics between molecular subtypes.

Characteristics	Total (n=215)	Luminal-like (n=126)	Basal-like (n=89)	*P*
Age (years)				0.47
*Median (IQR)*	67 (59-73)	68 (59-74)	66 (58-73)	
Gender				0.326
*Male*	141(65.6%)	86 (68.3%)	55 (61.8%)	
*Female*	74 (34.4%)	40 (31.7%)	34 (38.2%)	
Smoking history				**0.025**
*No*	146(67.9%)	78 (61.9%)	68 (76.4%)	
*Yes*	69 (32.1%)	48 (38.1%)	21 (23.6%)	
Tumor location				0.739
*Renal pelvis*	96 (44.7%)	59 (46.8%)	37 (41.6%)	
*Ureter*	65 (30.2%)	37 (29.4%)	28 (31.5%)	
*Multifocal*	54 (25.1%)	30 (23.8%)	24 (27.0%)	
Pathological T stage				**<0.001**
*Ta/T1*	78 (36.3%)	58 (46.0%)	20 (22.5%)	
*T2*	33 (15.3%)	26 (20.6%)	7 (7.9%)	
*T3*	94 (43.7%)	40 (31.7%)	54 (60.7%)	
*T4*	10 (4.7%)	2 (1.6%)	8 (9.0%)	
Tumor grade				**0.019**
*Low grade*	43 (20.0%)	32 (25.4%)	11 (12.4%)	
*High grade*	172(80.0%)	94 (74.6%)	78 (87.6%)	
Histological variant				**<0.001**
*No*	178(82.8%)	117(92.9%)	61 (68.5%)	
*Yes*	37 (17.2%)	9 (7.1%)	28 (31.5%)	
LVI				**0.001**
*No*	138(64.2%)	92 (73.0%)	46 (51.7%)	
*Yes*	77 (35.8%)	34 (27.0%)	43 (48.3%)	
Concomitant CIS				0.742
*No*	199(92.6%)	116 (92.1%)	83 (93.3%)	
*Yes*	16 (7.4%)	10 (7.9%)	6 (6.7%)	
Ki-67 index (%)				**0.001**
*Median (IQR)*	50 (30-70)	40 (20-60)	60 (40-80)	

Boldface indicates statistically signiﬁcant difference.

**Table 3:** Univariate and multivariate Cox regression analyses of RFS based on two-tier subtyping.

Variable	Univariate analysis		Multivariate analysis	
	HR (95% CI)	*P*	HR (95% CI)	*P*
Age	0.99 (0.97-1.02)	0.508	–	–
Smoking history	0.92 (0.57-1.49)	0.736	–	–
Gender	0.61 (0.37-1.02)	0.059	–	–
Concomitant CIS	0.74 (0.27-2.02)	0.553	–	–
Tumor location		0.19	–	–
*Ureter vs Renal pelvis*	1.06 (0.62-1.84)	0.829	–	–
*Multifocal vs Renal pelvis*	1.61 (0.94-2.77)	0.083	–	–
Subtype (Two-tier)	2.78(1.750-4.409)	**< 0.001**	1.93 (1.17-3.19)	**0.01**
Pathological T stage		**< 0.001**		**0.016**
*T2 vs Ta/T1*	2.10 (0.87-5.07)	0.099	1.43 (0.55-3.73)	0.459
*T3 vs Ta/T1*	4.86 (2.52-9.38)	**< 0.001**	2.26 (1.01-5.02)	**0.046**
*T4 vs Ta/T1*	20.78 (8.37-51.60)	**< 0.001**	5.74 (1.89-17.46)	**0.002**
LVI	3.97 (2.50-6.30)	**< 0.001**	2.00 (1.14-3.49)	**0.015**
Ki-67(%)	1.02 (1.01-1.03)	**< 0.001**	1.01 (1.00-1.02)	0.189
Tumor grade	3.65 (1.59-8.41)	**0.002**	0.82 (0.30-2.24)	0.704
Histological variant	1.78 (1.04-3.07)	**0.036**	0.95 (0.54-1.68)	0.852

Boldface indicates statistically signiﬁcant difference.

### RiskGroup stratification and prognostic analysis

**Figure 4:**
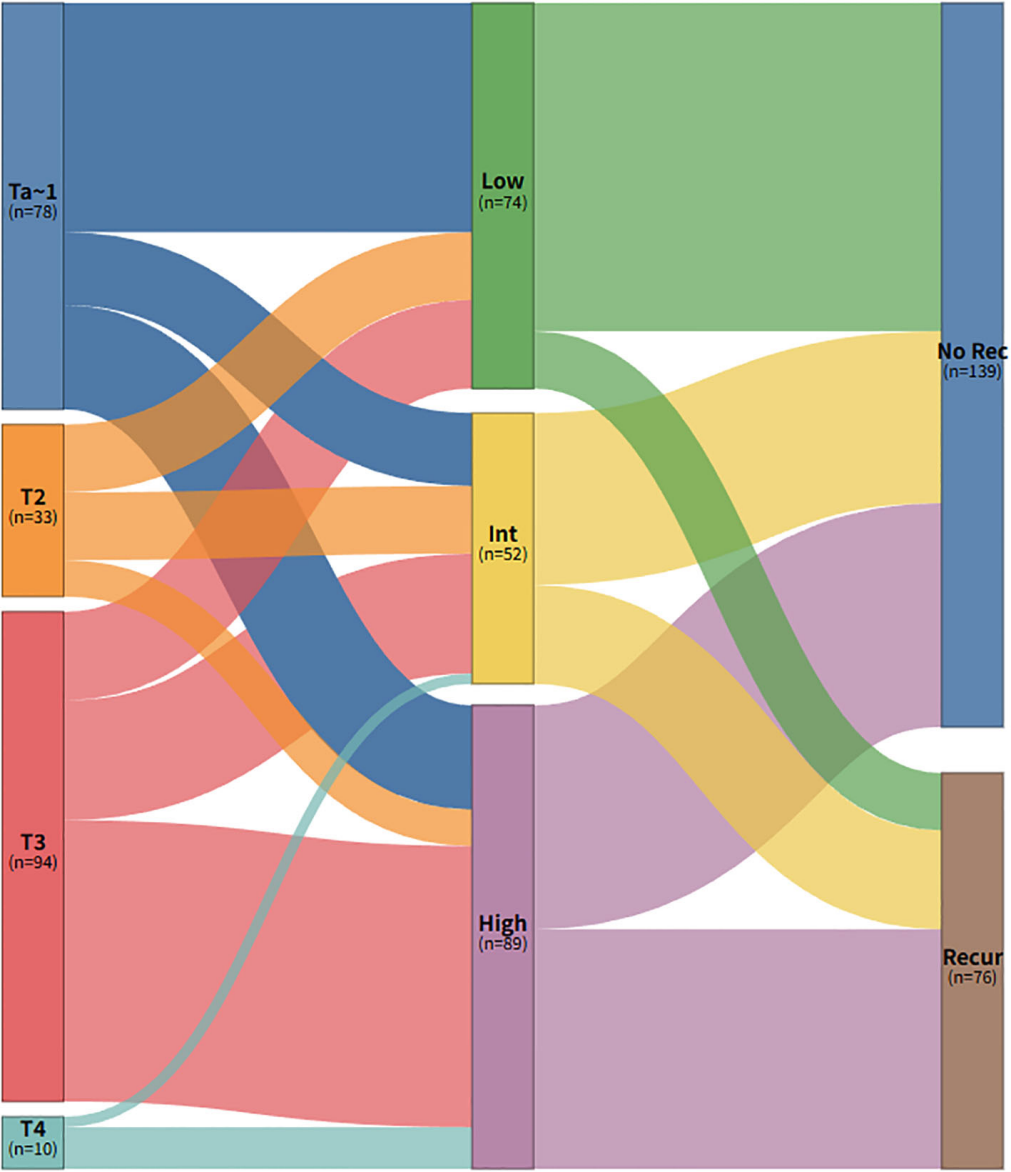
Sankey diagram illustrating the distribution of the RiskGroup stratification.

The integration of P53 status into the molecular subtyping framework (RiskGroup Stratification) unmasked significant biological heterogeneity within the cohort (**Table 4**). A distinct stepwise elevation in the Ki-67 proliferation index was observed across the Low, Intermediate, and High Risk groups (median 30%, 60%, and 60%, respectively; P < 0.001), reflecting a progressive dysregulation of the cell cycle. Furthermore, FGFR3 expression exhibited a significant inverse correlation with the risk hierarchy (P < 0.001). The highest positivity rate was observed in the Low Risk group (66.2%), which was significantly higher than that in the Intermediate Risk group (23.1%) and the High Risk group (14.6%). Strikingly, the Intermediate Risk group exhibited a paradoxical phenotype: while retaining the Luminal-like molecular background, it displayed aggressive histological features driven by P53 alterations. The proportion of high-grade tumors in this subgroup reached 94.2%, which showed no significant statistical difference compared to the High Risk group (87.6%; P = 0.207). Furthermore, compared to the Low Risk group, the Intermediate Risk group showed a significantly higher burden of locally advanced disease (pT3) (44.2% vs. 23.0%; P = 0.012) and LVI positivity (38.5% vs. 18.9%; P = 0.015) (**Figure 4**). The High Risk group (Basal-like) represented the most aggressive entity, characterized by the highest incidence of LVI (48.3%) and distant metastasis (37.1%).

**Table 4:** Comparison of clinicopathological characteristics among RiskGroup subtypes.

Variables	Low risk (n=74)	Int risk (n=52)	High risk (n=89)	P
Age (years),				
*Median (IQR)*	67 (58.0-73.0)	68 (60.3-74.8)	66 (58.5-73.0)	0.457
Gender				0.607
*Male*	51 (68.9%)	35 (67.3%)	55 (61.8%)	
*Female*	23 (31.1%)	17 (32.7%)	34 (38.2%)	
Smoking history				0.073
*No*	47 (63.5%)	31 (59.6%)	68 (76.4%)	
*Yes*	27 (36.5%)	21 (40.4%)	21 (23.6%)	
Pathological T stage				**< 0.001**
*Ta/T1*	44 (59.5%)	14 (26.9%)	20 (22.5%)	
*T2*	13 (17.6%)	13 (25.0%)	7 (7.9%)	
*T3*	17 (23.0%)	23 (44.2%)	54 (60.7%)	
*T4*	0 (0.0%)	2 (3.8%)	8 (9.0%)	
Tumor grade				**< 0.001**
*Low Grade*	29 (39.2%)	3 (5.8%)	11 (12.4%)	
*High Grade*	45 (60.8%)	49 (94.2%)	78 (87.6%)	
Concomitant CIS				0.79
*No*	69 (93.2%)	47 (90.4%)	83 (93.3%)	
*Yes*	5 (6.8%)	5 (9.6%)	6 (6.7%)	
LVI				**< 0.001**
*No*	60 (81.1%)	32 (61.5%)	46 (51.7%)	
*Yes*	14 (18.9%)	20 (38.5%)	43 (48.3%)	
Histological variant				**< 0.001**
*No*	69 (93.2%)	48 (92.3%)	61 (68.5%)	
*Yes*	5 (6.8%)	4 (7.7%)	28 (31.5%)	
Tumor location				0.145
*Renal pelvis*	40 (54.1%)	19 (36.5%)	37 (41.6%)	
*Ureter*	22 (29.7%)	15 (28.8%)	28 (31.5%)	
*Multifocal*	12 (16.2%)	18 (34.6%)	24 (27.0%)	
Recurrence Site				**< 0.001**
*Bladder recurrence only*	8 (72.7%)	10(52.6%)	13(28.3%)	
*Others*	3(27.3%)	9(47.4%)	33(71.7%)	
FGFR3				**< 0.001**
*Positive*	49(66.2%)	12(23.1%)	13(14.6%)	
*Negative*	25(33.8%)	40(76.9%)	76(85.4%)	
Ki-67 index (%)				
*Median (IQR)*	30 (15.0-50.0)	60 (40.0-70.0)	60 (30.0-70.0)	**< 0.001**

Boldface indicates statistically signiﬁcant difference.

**Figure 5:**
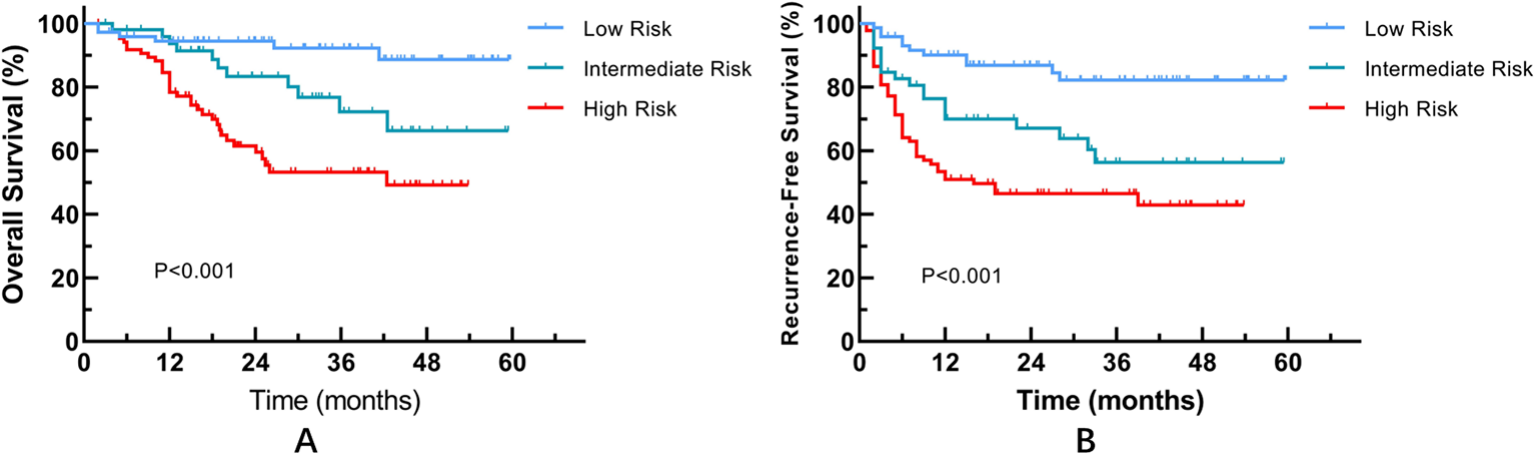
Kaplan-Meier survival curves based on the RiskGroup stratification. **(A)** Overall Survival (OS); **(B)** Recurrence-Free Survival (RFS).

Kaplan-Meier survival analysis revealed significant risk stratification for Recurrence-Free Survival (RFS) among the three groups (Log-rank P < 0.001) (**Figure 5**). The Low Risk group exhibited the most favorable prognosis, followed by the Intermediate Risk group, while the High Risk group showed the poorest outcomes (mean RFS: 27.8 months). In the multivariate Cox regression analysis (**Table 5**), using the Low Risk group as the reference, the High Risk group emerged as a robust independent predictor of RFS (HR = 2.600, 95% CI: 1.27–5.33, P = 0.009). The Intermediate Risk group also showed an elevated hazard ratio (HR = 1.63); however, this did not reach statistical significance after adjusting for tumor grade and stage (P = 0.227).

**Table 5:** Univariate and multivariate Cox regression analyses of RFS based on RiskGroup stratification.

Variable	Univariate analysis		Multivariate analysis	
	HR (95% CI)	P	HR (95% CI)	P
Age	0.99 (0.97-1.02)	0.508	–	–
Gender	0.61 (0.37 - 1.02)	0.059	–	–
Smoking history	0.92 (0.57-1.49)	0.736	–	–
Tumor location		0.19	–	–
*Ureter vs Renal pelvis*	1.06 (0.62-1.84)	0.829	–	–
*Multifocal vs Renal pelvis*	1.61 (0.94-2.77)	0.083	–	–
Pathological T stage		**< 0.001**		**0.016**
*T2 vs Ta/T1*	2.10 (0.87-5.07)	0.099	1.42 (0.55-3.69)	0.471
*T3 vs Ta/T1*	4.86 (2.52-9.38)	**< 0.001**	2.21 (1.00-4.91)	0.051
*T4 vs Ta/T1*	20.78 (8.37-51.60)	**< 0.001**	5.71 (1.88-17.33)	**0.002**
Tumor grade	3.65 (1.59 - 8.41)	**0.002**	0.86 (0.32 - 2.32)	0.759
Concomitant CIS	0.74 (0.27 - 2.02)	0.553	–	–
LVI	3.965 (2.50-6.30)	**< 0.001**	1.96 (1.12-3.43)	**0.019**
Histological variant	1.78 (1.04-3.07)	**0.036**	0.95 (0.54-1.68)	0.867
Ki-67 (%)	1.02 (1.01-1.03)	**< 0.001**	1.01 (0.99-1.02)	0.34
RiskGroup		**< 0.001**		**0.021**
*Int vs Low Risk*	2.76 (1.31-5.80)	**0.007**	1.63 (0.74- 3.61)	0.227
*High vs Low Risk*	4.67 (2.41-9.03)	**< 0.001**	2.60 (1.27-5.33)	0.009

Boldface indicates statistically signiﬁcant difference.

### Construction and validation of the prognostic nomogram

**Figure 6:**
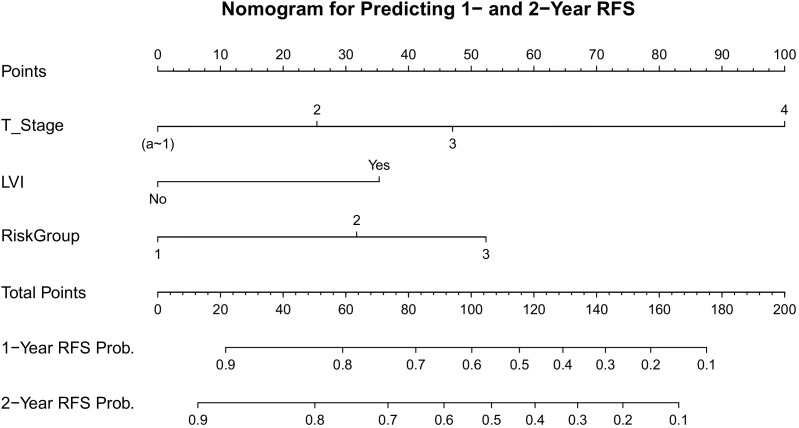
Nomogram for predicting 1-year and 2-year postoperative tumor recurrence.

**Figure 7:**
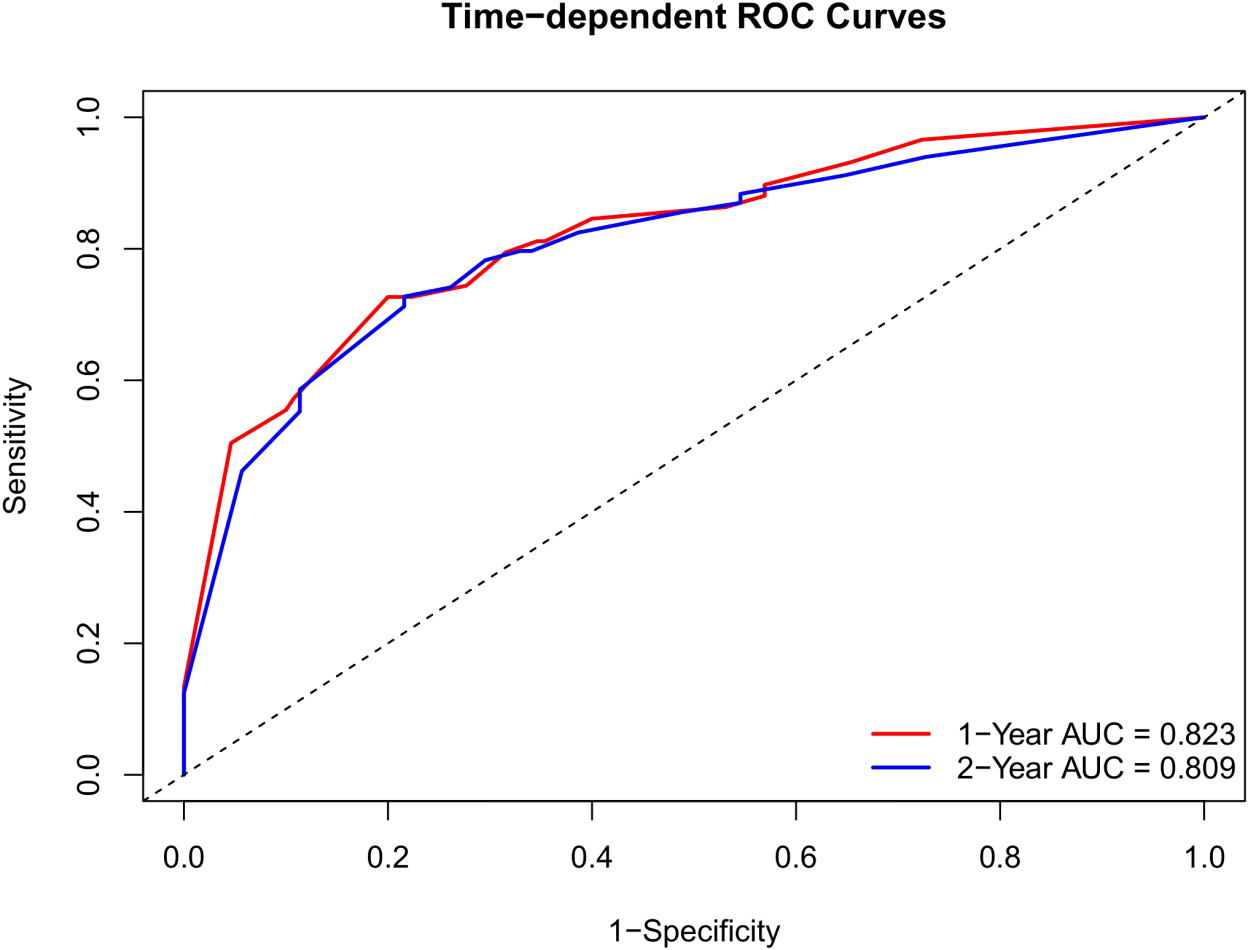
Time-dependent ROC curves of the nomogram for predicting 1-year and 2-year tumor recurrence.

**Figure 8:**
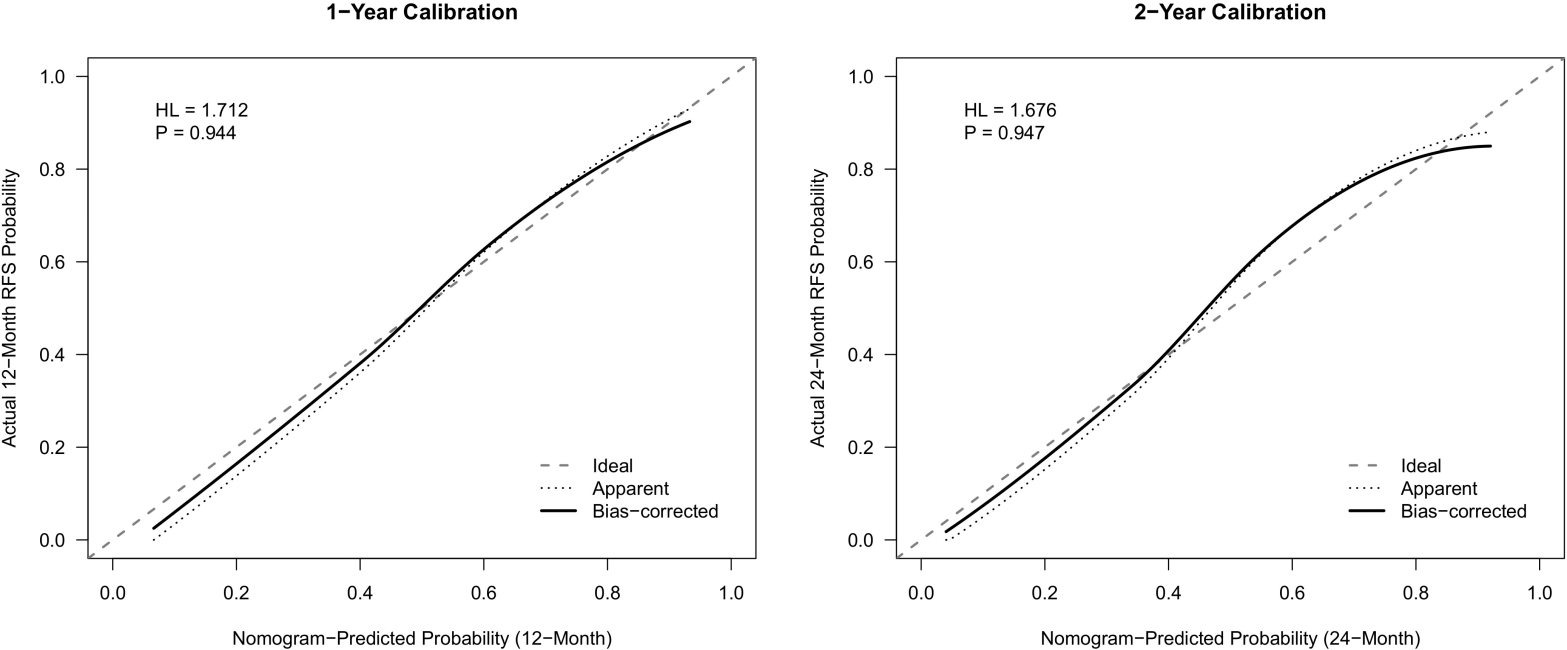
Calibration curves of the nomogram for predicting 1-year and 2-year tumor recurrence.

**Figure 9:**
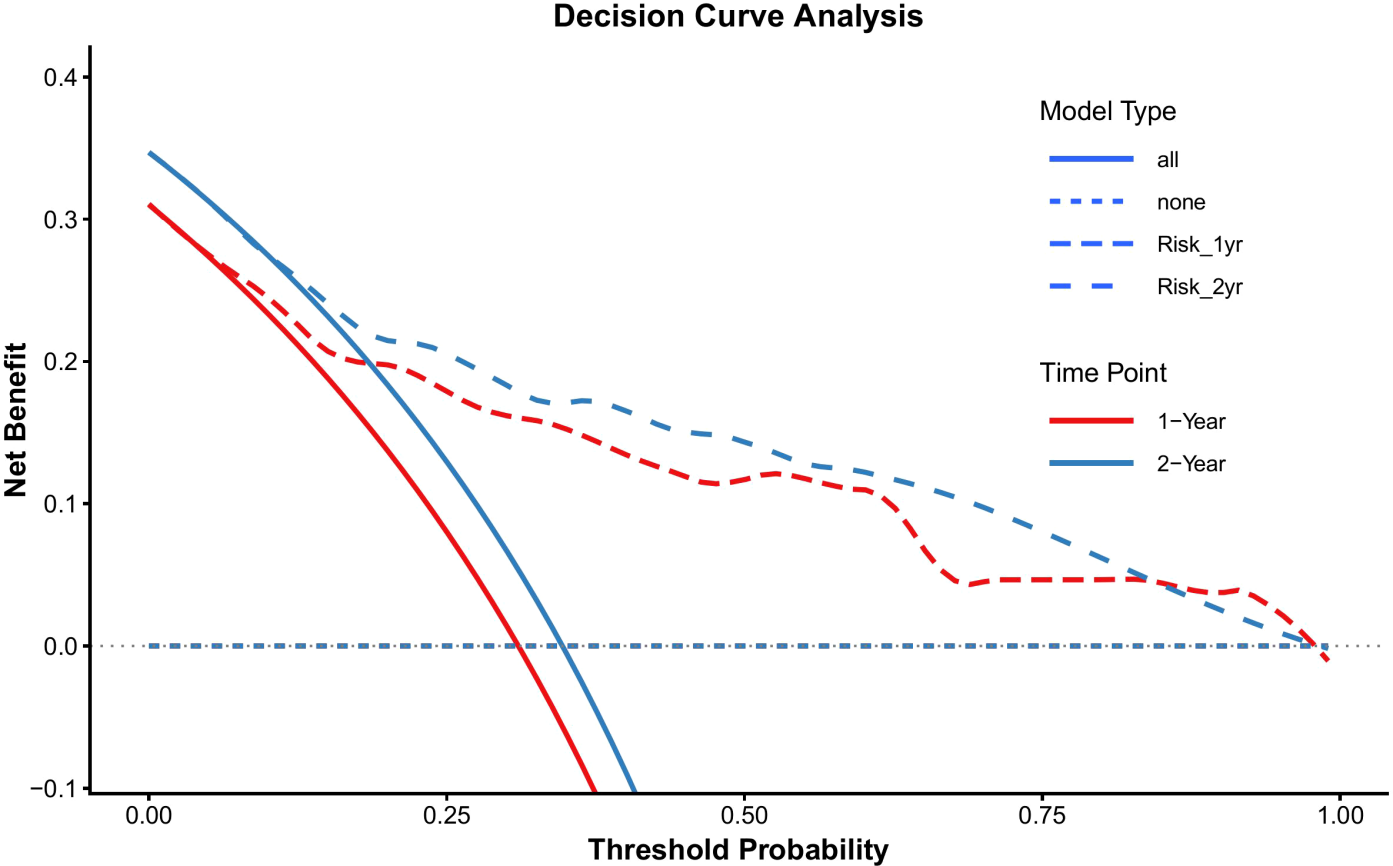
Decision Curve Analysis (DCA) of the nomogram for predicting 1-year and 2-year tumor recurrence.

To facilitate the clinical translation of our findings, a prognostic nomogram was established to predict 1- and 2-year Recurrence-Free Survival (RFS) (**Figure 6**). This model integrated the three independent risk factors identified in the multivariate analysis: RiskGroup, Pathological T stage, and Lymphovascular Invasion (LVI). Internal validation using the Bootstrap method (1,000 resamples) demonstrated that the nomogram possessed robust discriminatory power, achieving a Concordance Index (C-index) of 0.769 (95% CI: 0.713–0.823). Time-dependent ROC analysis (**Figure 7**) further confirmed its predictive accuracy, with an Area Under the Curve (AUC) of 0.823 for predicting 1-year RFS and 0.809 for 2-year RFS. Notably, the model exhibited a high Negative Predictive Value (NPV) of 88.1% for 1-year recurrence, indicating its reliability in identifying patients at low risk of early failure (**Table 6**). Calibration curves revealed a high degree of consistency between the nomogram-predicted probabilities and the actual observed recurrence rates (**Figure 8**). Furthermore, Decision Curve Analysis (DCA) demonstrated substantial net clinical benefit (**Figure 9**). Using the nomogram to guide clinical decision-making yielded superior benefit compared to “treat-all” or “treat-none” strategies across a broad threshold probability range. Of note, this single-center cohort was enriched for high-grade and locally advanced tumors, which may inflate apparent model performance and limits generalizability to lower-risk UTUC populations.

**Table 6:** Predictive performance of the nomogram for RFS.

Nomogram	AUC(95%CI)	Cut-off value	Sensitivity	Specificity	Youden index	PPV	NPV
1-year RFS	0.82(0.76~0.89)	0.20	0.73	0.80	0.53	0.59	0.88
2-year RFS	0.80(0.74~0.88)	0.25	0.73	0.78	0.51	0.64	0.84

AUC, area under the receiver operating curves; CI, conﬁdence interval; NPV, negative predictive value; PPV, positive predictive value.

## Discussion

The landscape of urothelial carcinoma diagnosis and treatment has been fundamentally transformed by the advent of high-throughput sequencing. Pioneering genomics research, represented by The Cancer Genome Atlas (TCGA), has propelled the field into the era of molecular subtyping, elucidating the intrinsic biological distinctions in mutational profiles and chemosensitivity between Luminal and Basal subtypes (7). Building on this, Fujii et al. (19) further mapped the precise genomic landscape of UTUC, confirming a robust correlation between molecular subtypes and clinical prognosis. However, despite the granular insights provided by whole-exome sequencing (WES) or RNA-seq, their routine clinical implementation is significantly hindered by prohibitive costs and technical barriers. While FFPE material is increasingly utilized for NGS, the potential for DNA/RNA degradation in archival blocks remains a challenge compared to fresh frozen tissue. Furthermore, the complexity of bioinformatic interpretation requires specialized personnel and computational infrastructure, which are currently unavailable in many standard pathology departments (20). To bridge this translational gap, studies by Lerner et al. (21) and Dadhania et al. (15) have demonstrated that simplified subtyping models based on protein expression possess prognostic utility comparable to transcriptome-based classifications. Motivated by this rationale, our study retrospectively analyzed clinicopathological data from 215 patients with UTUC to explore a precise, accessible risk stratification system based on protein levels, aiming to provide a practical tool for clinical decision-making.

In our study, we first validated the feasibility of using a streamlined panel of four core antibodies—GATA3, CK20, CK5/6, and CD44—to classify UTUC into Luminal-like and Basal-like subtypes. Our results confirmed that this accessible IHC panel could effectively delineate tumor subgroups with distinct biological behaviors, showing high concordance with molecular classification models established in previous bladder cancer studies (15, 22). *GATA3*, a zinc-finger transcription factor, acts as a master regulator of urothelial terminal differentiation; its robust expression typically signifies that tumor cells retain their intrinsic urothelial lineage program and have not undergone significant dedifferentiation (23). In stark contrast, Basal-like tumors were defined by the expression of CK5/6 and CD44. This immunophenotype was confirmed by Choi et al. (24) to phenocopy the gene expression profile of triple-negative breast cancer, implying enriched stemness and epithelial-mesenchymal transition (EMT) potential. Within the Basal-like population, the proportion of locally advanced tumors (≥ pT3) was significantly elevated, and the incidence of LVI reached 48.3%, nearly double that of the Luminal-like subtype (27.0%; P = 0.001). This explains the precipitous drop observed in the survival curves for patients with this subtype, a finding that aligns with Sikic et al. (25), who identified the CK5/6(+)/CK20(-) pattern as a potent predictor of mortality. However, while this “two-tier system” captures the broad molecular landscape, our survival analysis revealed that the prognosis within the Luminal-like group was not uniform. Approximately 20% of patients with an apparently “benign” Luminal phenotype experienced early recurrence or progression.

To address the inability of the two-tier system to resolve “Luminal heterogeneity,” we refined our stratification by integrating the protein expression status of two core driver genes—*FGFR3* and *TP53*—to construct the three-tier RiskGroup model. This strategy is rooted in the “dual-track” oncogenic hypothesis described by Catto et al. (26), which posits that urothelial carcinoma evolves via two distinct molecular pathways: an FGFR3-driven, prognosis-favorable papillary pathway and a TP53-driven, prognosis-poor invasive pathway. Our proteomic data support this classical hypothesis: the Low Risk group (Luminal-like/P53) exhibited a distinct co-occurrence of high FGFR3 expression and wild-type P53, coupled with a remarkably low median Ki-67 index of 30%, therefore making them a candidate population for FGFR inhibitor therapy (27). Crucially, while most molecular classifications are derived from Western cohorts, Shang et al. (28) recently mapped the genomic landscape of Chinese UTUC patients. They found that although specific mutation frequencies (e.g., *HRAS*) may vary across ethnicities due to environmental exposures (e.g., aristolochic acid), *TP53* mutation remains the universal “molecular switch” driving progression to an invasive phenotype. Furthermore, given that Mercader et al. (29) validated FGFR3 overexpression as a highly sensitive surrogate for *FGFR3* genomic alterations, our definition of the Low Risk group as “FGFR3-driven” is biologically robust.

A pivotal achievement of our risk stratification strategy is the identification of the Intermediate Risk subgroup. Phenotypically, these patients present a confounding profile: they retain the Luminal immunophenotype characteristic of differentiated tumors, yet molecularly, they harbor aberrant P53 expression (diffuse strong positivity or null type). In a conventional two-tier system relying solely on GATA3/CK5/6, these patients are prone to be misclassified as “low risk,” potentially leading to undertreatment. However, our clinicopathological analysis unmasks their aggressive nature: despite luminal differentiation, the median Ki-67 index in this group escalated to 60% (P < 0.001 vs. Low Risk), accompanied by higher pathological T stage (P = 0.002), tumor grade (P < 0.001), and LVI rates (P = 0.01). This resulted in significantly shorter OS (P = 0.02) and RFS (P < 0.01) in survival analysis compared to the low-risk group. Similarly, the study by Senol et al. (30) confirmed that abnormal p53 expression is significantly associated with high invasiveness and poor survival in urothelial carcinoma (P = 0.032). A study by Nagata et al. (16) demonstrated that urothelial carcinoma with abnormal p53 expression responds significantly to Enfortumab Vedotin (EV) treatment, with the objective response rate (ORR) and disease control rate (DCR) being significantly higher in patients with abnormal p53 than in those with wild-type p53 (p = 0.038 and p = 0.033, respectively).

Finally, we defined the High Risk group as Basal-like tumors characterized by the robust expression of CK5/6 and CD44. Multivariate Cox regression analysis confirmed that the recurrence risk in the High Risk group was more than twofold that of the Low Risk group (HR > 2.0), correlating with a high LVI positivity rate of 48.3% and a median Ki-67 index soaring to 60%. This subgroup accurately captures the “Basal/Squamous” entity described by Kamoun et al. (31), which, despite accounting for only 15%–25% of urothelial carcinomas, is characteristically enriched in the abnormal activation of EGFR, HIF-1α, and STAT3 signaling pathways. These pathways collectively drive rapid tumor proliferation and the formation of a hypoxic microenvironment. According to Helal et al. (32), this subtype represents a potential beneficiary of EGFR-directed therapy, however, it frequently demonstrates resistance to platinum-based chemotherapy (33). Notably, while Yang et al. (11) reported based on genomic sequencing that the overall *TP53* mutation rate in Chinese UTUC is lower than in bladder cancer, our data revealed that P53 mutant-type expression was highly prevalent (68.5%) within this specific Basal-like subset. In the present study, P53 status was not utilized as a basis for further stratification of the high-risk group. This is because, according to previous research (31), the dedifferentiated state represented by strong CK5/6 and CD44 positivity inherently predicts a dismal prognosis, and further subdivision would offer limited incremental clinical benefit.

The RiskGroup stratification constructed herein not only elucidates the biological heterogeneity of UTUC at the protein level but also demonstrates robust internal consistency with key clinicopathological parameters, supporting the concept that molecular phenotype is closely linked to clinical behavior. Our analysis revealed a striking “stepwise” progression in tumor grade, stage, and proliferative activity across the risk subtypes. While low-grade tumors were prevalent in the Low Risk group (39.2%), the proportion of high-grade tumors surged to 94.2% in the Intermediate Risk group and remained elevated at 87.6% in the High Risk group (*P < 0.001*). This deterioration in pathological phenotype was further quantified by the cell proliferation marker Ki-67: the median index climbed from 30% in the Low Risk group to 60% in the Intermediate Risk group, matching the high proliferation level (60%) observed in the High Risk group. Krabbe et al. (34) established that Ki-67 overexpression (>20%) is an independent predictor of decreased disease-specific survival (DSS) in high-grade UTUC. In our cohort, Ki-67 levels in both the Intermediate and High Risk groups consistently exceeded this alert line, signaling a substantial risk of early postoperative recurrence. Furthermore, the differential distribution of lymphovascular invasion (LVI)—a prerequisite for metastasis—carries profound clinical implications. The LVI positivity rate in the High Risk group was 48.3%, approximately 2.5-fold that of the Low Risk group (18.9%), with a distant metastasis rate reaching 37.1%, confirming its strong invasiveness and systemic dissemination potential. This reasonably explains why patients with Basal-type UTUC are clinically more prone to occult lymph node metastasis, leading to higher rates of postoperative recurrence and metastasis.

Translating molecular subtyping into clinically applicable tools is a key objective of this study, and the proposed RiskGroup system may have practical value. In multivariable analysis, RiskGroup remained a strong correlate of recurrence-free survival (RFS) after adjustment for major pathological confounders, including pathological T stage, tumor grade, and lymphovascular invasion (LVI). These findings support the feasibility of RiskGroup as a postoperative prognostic indicator for UTUC, providing biologically informed complementary information beyond standard clinicopathological risk stratification. At the same time, by integrating key features of established molecular subtyping through an IHC-based framework, RiskGroup may provide a pragmatic framework for future studies of treatment stratification in the evolving era of precision therapy for urothelial carcinoma (35). However, due to the lack of complete postoperative treatment data in the present study, the significance of RiskGroup for treatment decision-making could not be further evaluated. Nonetheless, RiskGroup showed a clear and biologically coherent association with established clinicopathological variables. Consistent with other similar studies (32), as the subtype shifted, tumor grade, extent of local invasion, incidence of LVI, and proliferative activity all worsened in a stepwise manner, suggesting that this classification does not merely represent a statistical grouping independent of pathology, but rather reflects a biological continuum consistent with tumor dedifferentiation and increasing invasiveness. Given these biological interrelationships, some degree of redundancy is expected even when formal multicollinearity is not evident (VIF < 2). Consistent with this, the high-risk category retained an independent association with RFS, whereas the intermediate-risk category did not remain statistically significant after adjustment (P = 0.227), indicating that its prognostic information is partially shared with conventional pathology rather than fully incremental. Collectively, these results support the RiskGroup as complementary to standard pathology-based risk stratification.

The prognostic nomogram constructed based on these variables exhibited favorable discrimination in our cohort, achieving a Bootstrap-validated C-index of 0.769 (95% CI: 0.713–0.823). Furthermore, Decision Curve Analysis (DCA) suggested that using this nomogram may offer a positive net benefit over default strategies across a range of threshold probabilities, highlighting its potential as a supplementary tool for post-operative risk assessment. By combining RiskGroup with pathological T stage and lymphovascular invasion (LVI), the model enables individualized assessment of recurrence risk in patients after radical nephroureterectomy. It may help identify patients at low risk of early recurrence, thereby avoiding unnecessarily intensive follow-up, while also helping to identify high-risk patients who may require closer cystoscopic, radiologic, and clinical surveillance. More broadly, the nomogram may also serve as a supportive framework for postoperative counseling and for future studies evaluating risk-adapted surveillance intensity or adjuvant management strategies. Of note, our study cohort is predominantly composed of high-pT stage and high-grade tumors. This overrepresentation of high-risk features may exaggerate the proportion of highly aggressive tumors within each risk stratum or artificially inflate the size of the high-risk group. Consequently, the model may overestimate recurrence risk in truly low-risk tumors and should therefore be interpreted cautiously when extrapolated to lower-risk UTUC populations. Furthermore, while our IHC-based RiskGroup is an independent prognosticator, traditional parameters like pT stage and LVI continue to drive the nomogram’s predictive weights. Overall, the nomogram should be interpreted as an exploratory, hypothesis-generating tool that requires external validation in independent, preferably multicenter cohorts spanning the full clinical risk spectrum before it can be considered for clinical implementation.

This study has several limitations. First, it is a single-center retrospective analysis and lacks external validation. Second, the cohort is enriched for high-grade and locally advanced tumors, which may overestimate the model’s discriminatory performance and limits extrapolation to lower-risk UTUC populations. Third, IHC provides an accessible surrogate for underlying genomic alterations; however, protein expression can be influenced by non-mutational mechanisms and is not fully equivalent to sequencing-based assessments. Therefore, further validation integrating multi-omics data is warranted. Finally, due to incomplete data on postoperative systemic therapy, we were unable to evaluate whether RiskGroup has predictive value for treatment response.

In conclusion, a simplified IHC panel (GATA3, CK20, CK5/6, and CD44) enables effective dichotomization of UTUC into luminal-like and basal-like subtypes with distinct biological behaviors. Incorporating p53 status, the proposed RiskGroup further stratifies patients into three prognostically distinct subgroups. The resulting nomogram shows promising performance for postoperative RFS prediction. Nonetheless, because this cohort is predominantly high-risk and treatment-response prediction could not be evaluated, independent external validation and further investigation are warranted.

## Data Availability

The raw data supporting the conclusions of this article will be made available by the authors, without undue reservation.
